# The motivation-based calving facility: Social and cognitive factors influence isolation seeking behaviour of Holstein dairy cows at calving

**DOI:** 10.1371/journal.pone.0191128

**Published:** 2018-01-18

**Authors:** Maria Vilain Rørvang, Mette S. Herskin, Margit Bak Jensen

**Affiliations:** Aarhus University, Department of Animal Science, Tjele, Denmark; University of Illinois, UNITED STATES

## Abstract

In order to improve animal welfare it is recommended that dairy farmers move calving cows from the herd to individual pens when calving is imminent. However, the practicality of moving cows has proven a challenge and may lead to disturbance of the cows rather than easing the process of calving. One solution may be to allow the cow to seek isolation prior to calving. This study examined whether pre-parturient dairy cows will isolate in an individual calving pen placed in a group calving setting and whether a closing gate in this individual calving pen will cause more cows to isolate prior to calving. Danish Holstein cows (n = 66) were housed in groups of six in a group pen with access to six individual calving pens connected to the group area. Cows were trained to use one of two isolation opportunities i.e. individual calving pens with functional closing gates (n = 35) allowing only one cow access at a time, or individual calving pens with permanently open gates allowing free cow traffic between group area and individual pen (n = 31). The response variables were calving site, calving behaviour and social behaviour. Unexpectedly, a functional gate did not facilitate isolation seeking, perhaps because the cows were not able to combine a learnt response with the motivation to isolate. Dominant cows had the highest chance of calving in an individual calving pen. If an alien calf was present in the group pen or any of the individual pens, cows were less likely to calve in an individual calving pen. Future studies should allow cows easy access to an individual calving pen and explore what motivates pre-parturient cows to seek isolation in order to facilitate voluntary use of individual calving pens.

## Introduction

Calving is an essential part of commercial milk production and during the transition period (typically defined as three weeks before and three weeks after calving), there are several threats to dairy cow welfare. Calving itself places high demands physically and is associated with pain [1]. Disease and mortality rates are high during this period, with nearly 75% of disease cases occurring within the first month after calving (reviewed in [2]) and 30–50% of post-partum cows being affected [3]. When cows calve in group calving pens they are frequently disturbed by other cows and the risk of mismothering is high [4]. In order to protect the calving cow, it is currently recommended that cows calve in individual calving pens to which they are moved well in time before calving (e.g. by law in Denmark by Ministry of Environment and Food of Denmark, Danish Veterinary and Food Administration, Law number. 520, Chapter 4, 26/05/2010, [5] and by recommendation of The Canadian Dairy Code of Practice [6]). Cows are, however, often moved too late due to the difficulty of determining the onset of the first stages of labour, which have been suggested to be an appropriate time to move cows to individual calving pens [7,8]. Moving cows later may disturb the process of calving and prolong the second stage of labour [7], leading to an increased risk of calving complications (dystocia) and related diseases [9]. The current international trend towards increased herd size [10] means that farmers have more cows to supervise, making it more difficult to identify the correct time to move cows to individual calving pens. Increased herd size may also result in farmers using group calving facilities more often, which may not be consistent with the choice of the cows. In order to aid identification of imminent calving, devices have been developed to monitor behavioural and physiological changes before calving, such as reduced rumination [11] and increased number of lying bouts [12,13]. In addition, vaginal temperature has been reported to decrease prior to calving [14] and sensors to detect this physiological change are now available. However, these changes are only measurable in the hours prior to calving meaning that many cows are unlikely to be moved in time. Hence, there is a need for a practical solution facilitating the moving of cows well in time before calving. One possible solution may be to develop a motivation-based calving facility, taking advantage of the natural motivation of calving cows to seek isolation.

To design motivation-based calving facilities, knowledge of the behaviour and preferences of calving cows are essential. The natural behaviour of cattle is to stay within the proximity of the herd, and typically cattle synchronize their behaviour [15,16]. However, as parturition approaches, cows become restless [14,17,18] and move away from the group and isolation seeking behaviour have been reported among cows kept in semi-natural [19] and production conditions [20,21]. The underlying evolutionary strategy may be to ensure that the female bonds to her offspring without disturbance from group members, but isolation seeking behaviour may also have developed to protect the newborn against predation as suggested by [22]. In a practical setting, motivation for isolation seeking may be utilized when cows are to be moved into individual calving pens before calving, by allowing cows to move into these pens on their own, and at the same time ensure a timely move to individual housing, as well as ensuring an undisturbed environment.

One aspect of isolation seeking behaviour is the social factors inevitably arising from housing pregnant cows in groups. In addition, individual differences between cows are apparent. Although sparsely studied, dairy cows have different levels of sociability [23] and individual behavioural characteristics [24]. Making a choice of where to calve, may also depend on the personality of the cow. To our knowledge, neither the effects of social factors, nor personality, has been studied previously. Edwards [4] noted that when calving in groups, cows close to parturition were exceedingly attracted to calves (as 14 out of 16 cows licked an alien calf before giving birth themselves). Calves are, however, not the only distraction. In intensive indoor dairy production, pre-parturient cows are often moved to a new group, where the level of aggression can be relatively high [25], especially in relation to accessing limited resources, such as an individual calving pen. Adding to this, maternal aggression (expression of defensive and aggressive behaviour to protect the offspring [26]) is well documented in a number of ungulates (cattle: [27] sheep: [28], pigs: [29]), which may lead to even higher levels of aggression among peri-parturient females. Within a group of pre-parturient cows, social dominance determines the outcome of competition for resources [30]. If individual calving pens are perceived as a limited resource by the cows, the chance of gaining access to a pen is likely higher for dominant individuals. However, possible interactions between social dominance and stage of pregnancy cannot be ruled out. The motivation to seek isolation may also cause cows to avoid social confrontations and become more submissive as calving approaches. What happens in relation to personality and dominance in the pre-parturient cows and whether other factors affect the motivation to isolate before calving, must be determined in order to develop motivation-based calving facilities.

The aim of this study was to examine whether cows kept under conventional dairy farming conditions will isolate in an individual calving pen when they are trained to access this beforehand. We housed 13 groups of six pregnant dairy cows in group pens with access to individual calving pens, allowing either free cow traffic (the gate was permanently open) or access for only one cow at a time (functional mechanical gate). We hypothesized that cows housed with functional gates would be more likely to seek isolation in an individual calving pen prior to calving due to their experience of being alone, behind the closed gate compared to cows housed in groups where calving pens had free cow traffic and thus no gate to keep other cows out. Additionally, we investigated whether other factors, such as personality, dominance, group structure, and presence of alien calves influenced the choice of calving site during 12 to 8 h pre-calving. A personality assessment tests was incorporated into the experimental plan by using Human approach tests and recording social interactions. We hypothesized that subordinate cows would be more likely to seek isolation behind the gates due to increased risk of aggression from dominant cows and that the presence of an alien calf would lower the probability of cows moving into an individual calving pen.

## Materials and method

The experiment complied with the current Danish law, except for calving in individual calving pens and except for the cow-calf pairs being separated before 12 hours after calving. The procedures were evaluated prior to the experimental start by the responsible laboratory animal veterinarian from the institutional animal ethics committee at The Department of Animal Science, Aarhus University, Tjele, Denmark. It was confirmed that no ethical oversight was needed.

### Housing conditions

The experiment took place at the Danish Cattle Research facility at Aarhus University (Foulum, Denmark) September 2015 to June 2016.

**Fig 1:**
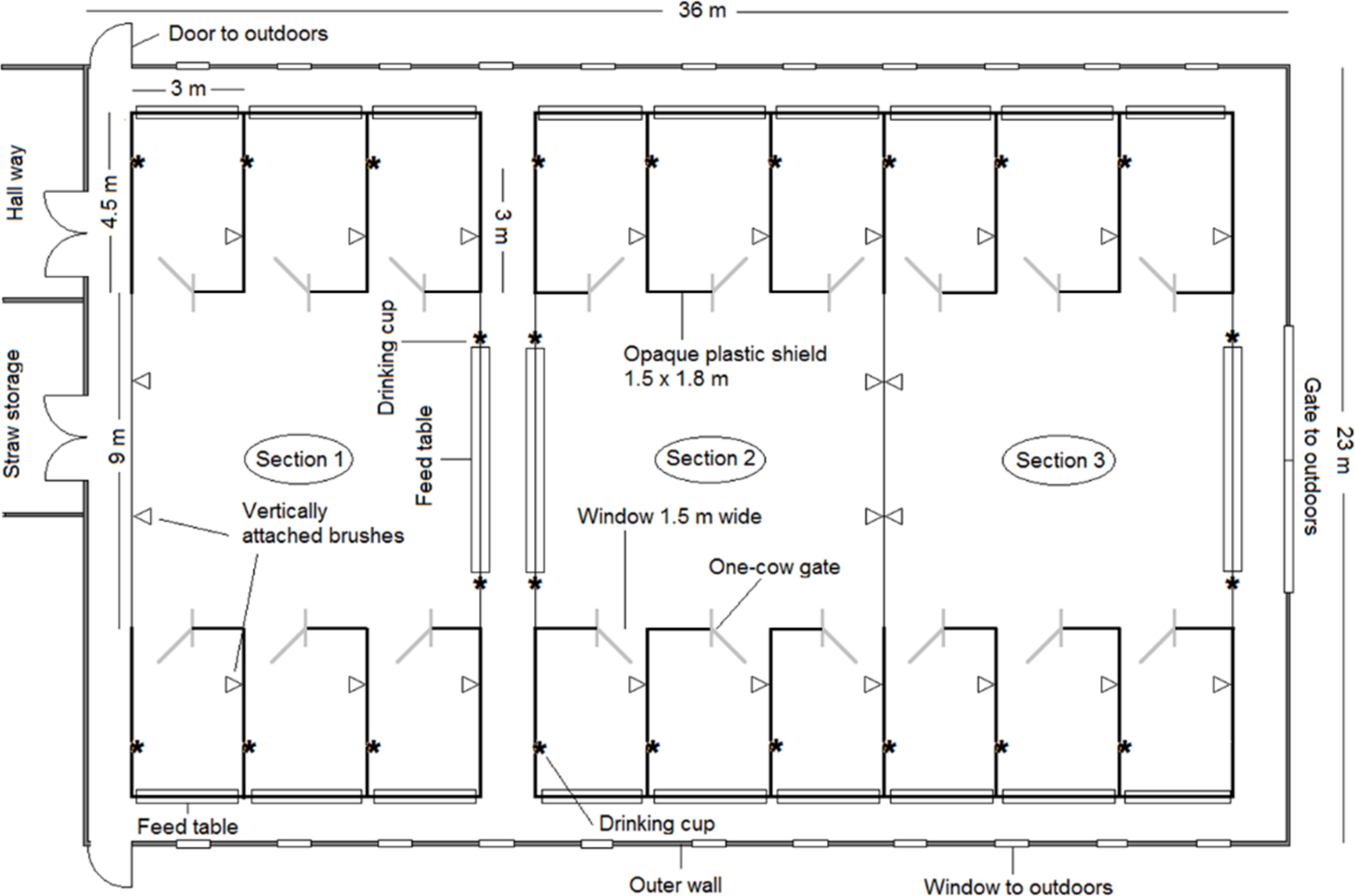
The experimental barn. Top view of the experimental barn, including all three sections. Thick lines around individual calving pens represent covered sides, and the “window” illustrates where the mechanical gate (grey insertion in the “window”) was installed in all individual calving pens. Vertically attached brushes, drinking cups and feed tables are shown.

**Fig 2:**
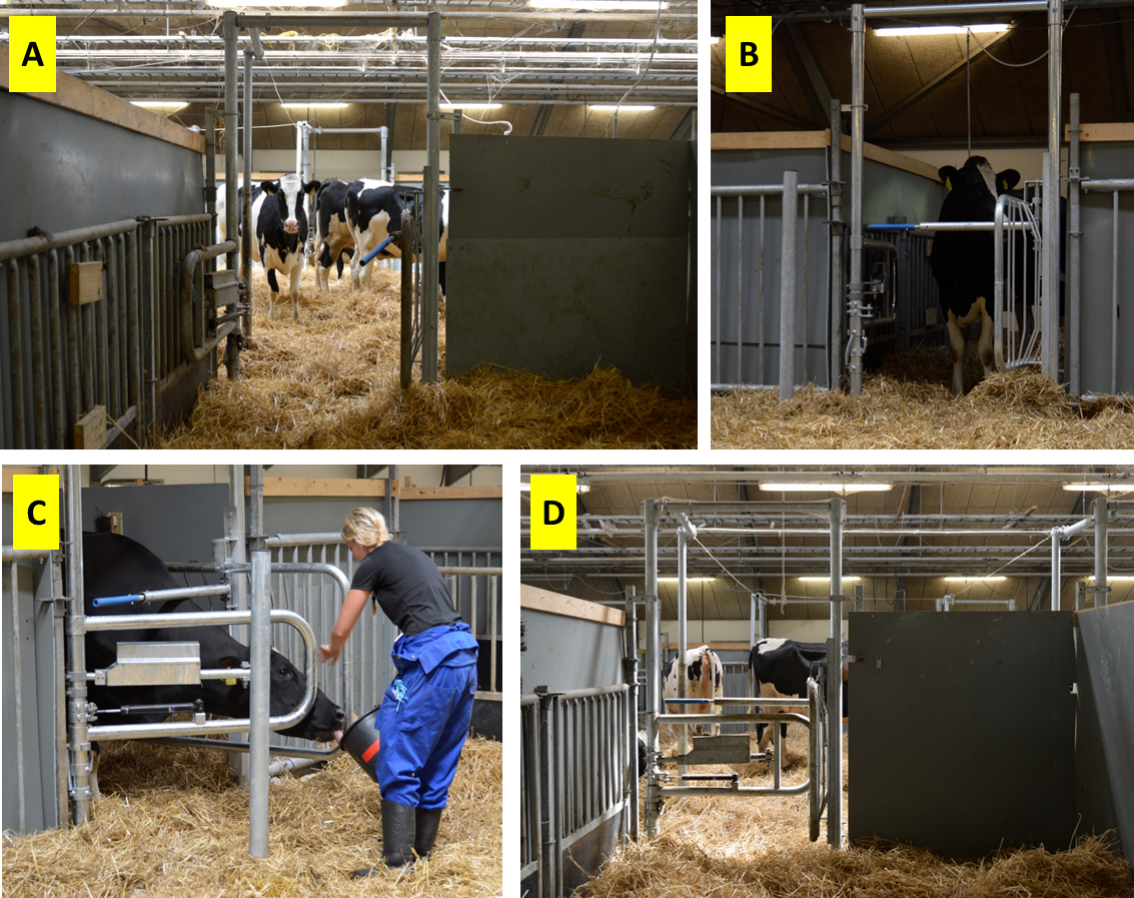
The levels of closing the gate. A: The view from an individual calving pen with open gate, representing the start point for all cows (initial training, *step 1*) and the treatment termed “permanently open gate”. B: The top bar of the gate being closed (corresponding to training *step 2* for cows housed with “functional gates”). C: The trainer holding the gate, half way open, in order for the cow to see the way out (corresponding to training *step 3* for cows housed with functional gates). D: The view from inside the gated individual calving pens with the gate fully closed (corresponding to training *step 4* for cows housed with “functional gates”).

The experimental barn had 3 sections (Fig 1), each consisting of a group area (9 x 9 m) connected to 6 adjacent individual calving pens (4.5 x 3 m each). The individual calving pens were built from 1.3 m high tubular metal bars covered by 1.8 m high grey plastic barriers (barrier width: 10mm, Jyden, Vemb, Denmark), on the two 4.5 m sides as well as 1.5 m of one other side (half of the pen side facing the group area), leaving a 1.5 m opening towards the group area. The fourth side (facing the outer walls of the barn) had a 3.0 m feed bunk (Jyden, Vemb, Denmark). This construction was chosen to offer the cows an opportunity to isolate (Fig 2A and 2D). In the opening into the group area (1.8 x 1.5 m), a mechanical cow gate was installed. The gate was designed to allow access for just one cow at a time by locking mechanically after one cow had entered the pen i.e. opened the gate and passed through (Prototype designed by Jyden, Vemb, Denmark). The cow was always able to go back to the group area, after which, the gate would unlock mechanically from the manipulation when the cow pushed the gate open, allowing another individual to enter. In half of the sections, the experimental calving pens were equipped with open gates (gate permanently open), while the other half of the sections had a functional gate (i.e. gate) (as illustrated in Fig 2A and 2D). The two experimental treatments (permanently open gate; functional gate) were applied to one whole section in a balanced order to control for possible effects of section placement within the barn. The order was (section; treatment: 1; permanently open, 2; functional, 3; functional, 1; functional, 2; permanently open, 3; permanently open, 1; functional, 2; permanently open, 3; functional, 1; permanently open, 2; functional, 3; permanently open, 1; functional).

The floor of all group areas and individual calving pens was covered by approximately 15 cm of sand (Kosand, brand; Dansand, Brædstrup, Denmark; mean grain size 0.322 mm) and approximately 15 cm of barley straw on top as bedding to ensure good lying comfort and a non-slip surface for getting up and lying down behaviour of the cows. New straw was added on a daily basis in the morning between 9.30 and 12.00 am after cleaning (removal of faeces and wet and soiled straw). Cleaning was done to maintain similar bedding quality in the different areas. After each calving, the place where the amniotic sac broke, and the place where the calf was born, was located using video surveillance (described below) and straw as well as sand was fully removed in an area of Ø: 1 m. Afterwards new sand and straw were added. This procedure was used in order to limit the potential influence from leftover birth fluids [31].

Each group area had 6 individual feed bins constituting the feed table (bin width: 75 cm, model 1318–8210, Jyden, Vemb, Denmark) and two self-filling, automatic drinking cups (model 2177–4010, Jyden, Vemb, Denmark). Each individual calving pen had one water cup similar to the ones in the group area.

Cows were only fed in the group area to avoid confounding of feed and isolation motivation in the individual calving pens. During the whole experimental period, a total mixed ration with a forage-to concentrate ratio of 80:20 (%, dry matter basis) was provided for ad libitum intake. Feed was allocated twice daily, in the morning between 9.30 and 12.00 am, and in the afternoon between 5.30 and 6.00 pm.

### Animals

Initially, the experiment included a total of 78 multiparous Danish Holstein cows all provided from the Danish Cattle Research facility at Aarhus University (Foulum, Denmark). This sample size was chosen based on a pre hoc power analysis, utilizing the probability of calving in a pen of 0.71 (found in a pilot experiment). Based on the power analysis, we decided to include 6 animals per section meaning that there was one individual pen per cow. The probability of detecting a difference in the experiment was 94% (at least 80% is recommended [32]) when including 72 animals. The total sample size thus consisted of 72 animals determined from the power analysis plus an additional 6 animals included as a buffer. The buffer of animals was included due to the possibility of having to exclude cows due to sickness, calving difficulty or other calving related issues (see exclusion criteria below).

Thirteen section groups were constructed based on expected calving dates in order to ensure a similar dispersion of calvings and allow barn staff time to clean between calvings. Section group cows had at least 1 day between expected calvings and the average dispersion between expected calvings was 6.9 days (range: 1 to 15 days).

### Training and tests

**Fig 3:**
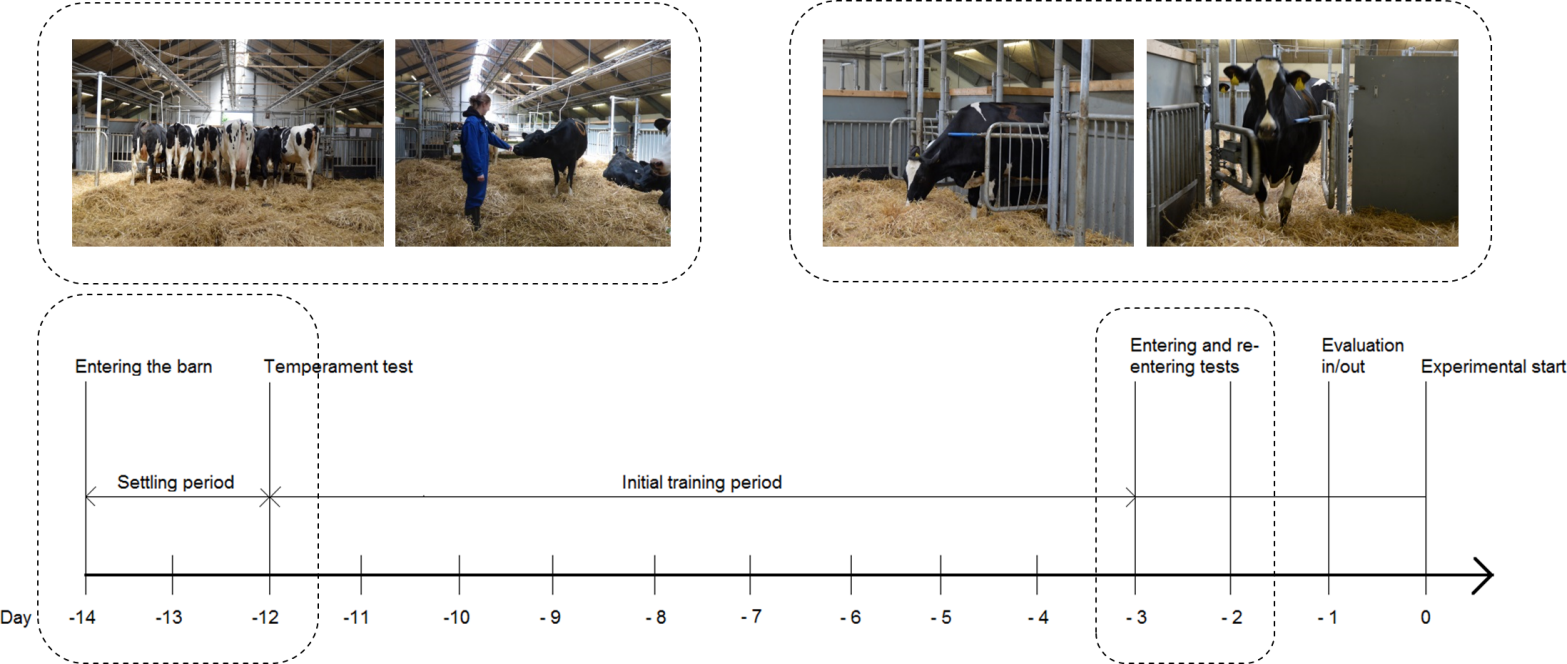
Time line. Days prior to experimental start, indicating all initial procedures and assessments. Moved to the barn (day -14), temperament test (day -12) after a settling period (day -14 to -12), initial training (day -12 to -3), entering and re-entering tests (day -3 and -2) leading to either inclusion or exclusion (day -1) and experimental start (day 0).

Each group of six cows was moved to the experimental section and placed in the group area approximately two weeks prior to the first expected calving. All cows were allowed 24 h to acclimatize to the experimental housing before initiation of training (Fig 3). During this period, all gates were kept permanently open regardless of treatment, and cows could move freely in and out of the individual calving pens. Depending on the gate treatment, a specific training procedure was used (see below).

#### Personality assessment

On the day of training initiation, a personality assessment of all cows was done based on the cows´ immediate reaction in terms of exploration-avoidance of the trainer and the training procedures. This was done in a “section entering test” and a “human approach test”, conducted prior to the initiation of training as well as during the first steps of the training procedures (described below) and termed “fearfulness during the initial training”. The “section entering test” assessed the cow’s immediate reaction to the trainer. When the trainer entered the barn for the first time, the trainer would stand in front of the group area for a few minutes (2 to 5 min) before entering the section. The trainer then climbed the outer walls and entered the middle of the group area. Inside the group area, the trainer walked to the centre, making sure that all cows paid attention, and then scored the cows according to the definitions shown in Table 1. The “human approach test” was initiated by identifying a cow in the group area not eating, drinking or engaging in social interactions, and which was attentive towards the trainer. The trainer would then approach the focal cow from a distance of two cow lengths (approximately 5 m) from an angle of 45° of the front of the cow, with an outstretched arm and an even pace (as described by [33]). When the cow moved away from the trainer, the avoidance distance was noted. If the cow did not move, the avoidance distance was left censored as zero (Table 1). The evaluation of “fearfulness towards the initial training” was carried out during the first training bout and consisted of evaluating the reaction of the cow towards the initial training procedure, and the level reached according to the initial training steps described below. Finally, each cow was evaluated as being “explorative” or “avoiding” according to the definitions in Table 1. A cow was determined ‘*shy’* when scored in the “avoiding” category for at least two out of three assessments, and likewise determined ‘*bold’* when scored in the “explorative” category for at least two out of three assessments. Boldness was thus defined as an explorative animal, i.e. willing to explore a novel person (the trainer) and a novel situation (the initiation of training) [34].

**Table 1:** Personality assessment.

Test	*Avoiding*	*Explorative*
*Section entering test*	Cow moves away from the trainer	Cow stands still or approaches the trainer
*Human approach test*	Avoidance distance more than one cow length from the trainer	Avoidance distance less than one cow length from the trainer
*Fearfulness during the initial training*	Started on *step 0* and reached no further than *step 1* in first training trial	Started on *step 1* and reached *step 1* or higher in the first training trial

Definition of the “Avoiding” and Explorative” categories according to the three assessments of the dairy cows done during the pre-experimental training period; Entering test, Human approach test and Fearfulness during the initial training. The cut-off for the avoidance distance in the Human approach test was determined before the test. A cow was determined ‘shy’ when scored in the “avoiding category” for at least two out of three assessments, and likewise determined ‘bold’ when scored in the “explorative category” for at least two out of three assessments. The initial training steps are described below.

#### Initial training

Cows were trained section-wise i.e., trained individually, but with the other five cows of the section present during training. Each training day had two distinct periods of training chosen based on expected level of feeding motivation and disturbances: 08 to 10 am just before feeding, and 01 to 03 pm just before topping up the feed.

Each training lasted a maximum of 8 minutes per cow (determined during a pilot training session with pilot cows prior to the experiment) and the average training duration was 4.8 ± 2.6 min per bout. Training bouts were separated by either one day or 3 to 7 hours. A training bout was always initiated from within the group area, and each cow was trained to enter a randomly chosen individual calving pen (i.e. decided beforehand by a third person rolling a dice). If the chosen individual calving pen was already occupied, the neighbouring pen to the right was chosen. Throughout the successive training bouts, cows were always trained in the pen to the right, during the proceeding training, in order to allow experience with all individual calving pens. No cow was chased out of an individual calving pen in order to be trained, i.e. if a cow was inside an individual calving pen, the trainer would wait until the cow re-entered the group area before initiating her training.

All cows were gradually trained to follow the trainer by reinforcing the approach by positive reinforcement i.e. eating concentrates from a black plastic bucket (12 litres). The trainer would stand in front of the cow and at first allow the cow to eat a mouthful from the bucket without having to move (*step 0*). If the cow was unwilling, or too fearful to eat from the bucket, she was allowed to eat from the bucket while it was standing on the bedding without the trainer present (watching from a 3 m distance). In such cases, the trainer would gradually approach the cow while she was eating from the bucket, until the trainer was able to touch and later lift the bucket. Shortly after completing *step 0*, the trainer moved a few steps away from the cow towards the individual calving pen while still facing the cow and displaying the bucket. When the cow followed, she was again allowed to eat a mouthful upon reaching the bucket and the trainer. At first, the reward would be given after just a few steps in order for the cow to learn to follow. The demand would then rise as training progressed. *Step 1* consisted of the trainer moving backwards through the opening of the individual calving pen, spending a few seconds inside the individual calving pen rewarding the cow and then leaving. When the cow walked willingly through the open gate three times, she had completed *step 1*.

Cows housed with functional gates *(n = 35)*: The gate was gradually closed in three steps: First the top bar was closed (*step 2*), secondly, the gate was closed, but the trainer held the gate halfway open in order for the cow to see the way in and out (*step 3*) and lastly, the gate was fully closed (*step 4*) (Fig 2B, 2C and 2D, respectively). In these steps, the trainer passed through the gate opening, and then faced the cow to entice her to pass. Each time the degree of closing the gate was changed, the procedure of going in and out was repeated until the cow walked willingly through, three times. During the training, the trainer ensured that no other cows followed the focal cow into an individual calving pen at any point. The success criterion for the initial training was that the cow should be able to open the closed gate on her own three times (i.e. two times going into the individual calving pen, and one time going out) without showing any fear-related behaviour (Table 2). For visual of cows being trained to perform step 4, see S1 Video.

**Table 2:** Description of fear-related behaviour to be absent when evaluating all trained cows irrespective of treatment according to their success criterion.

*Behaviour*	*Description*
Immobilization	The absence of movement of any limb or head[35]
Moving backwards	The animal is moving backwards by lifting and changing position of both forelegs or all four legs[36]
Flight	The animal runs (and/or jumps) at least 2 meters away from the trainer
Vigilance	Head raised above shoulder height, looking over barrier or side of the pen while upright, ears pointing forwards the direction of the head/eyes

None of the above behaviours could be shown during the last training step (i.e. step 1 or 4 respectively) if the cow were to comply with her criterion.

Cows housed with permanently open gates *(n = 31)*: All cows were gradually trained to walk with the trainer through the gate opening of the individual calving pen and back again, as described above (*step 1*). The duration of a training bout was determined as the mean duration of a corresponding training bout for cows trained to the functional gates. Likewise, the number of training bouts corresponded to the number of training bouts for cows trained to functional gates, and thus all cows, irrespective of the treatment, were trained for the same duration of time. In this treatment, other cows were allowed to follow the trained cow when she entered an individual calving pen, and likewise allowed to enter the individual calving pen with the focal cow and the trainer. The success criterion for this training was that the cow should be able to enter the individual calving pen (two times) and re-enter the group area (one time) without showing any fear-related behaviour (Table 2).

#### Entering- and re-entering tests

When a cow complied with either success criterion, she was left inside the individual calving pen to which she was trained as the trainer left through the gate. The cow then had a maximum of 4 hours to re-enter the group area on her own. This test was called *the re-entering test*.

When *the re-entering test* was passed, each cow was observed for 48 hours (on video) to check if she entered any individual calving pen(s) on her own. This period constituted *the entering test*.

When *initial training*, *the re-entering* as well as the *entering test* was passed successfully, a cow was considered ready for the experiment and thus complied with the overall success criterion (Fig 3).

#### Training progress and exclusion during training

**Fig 4:**
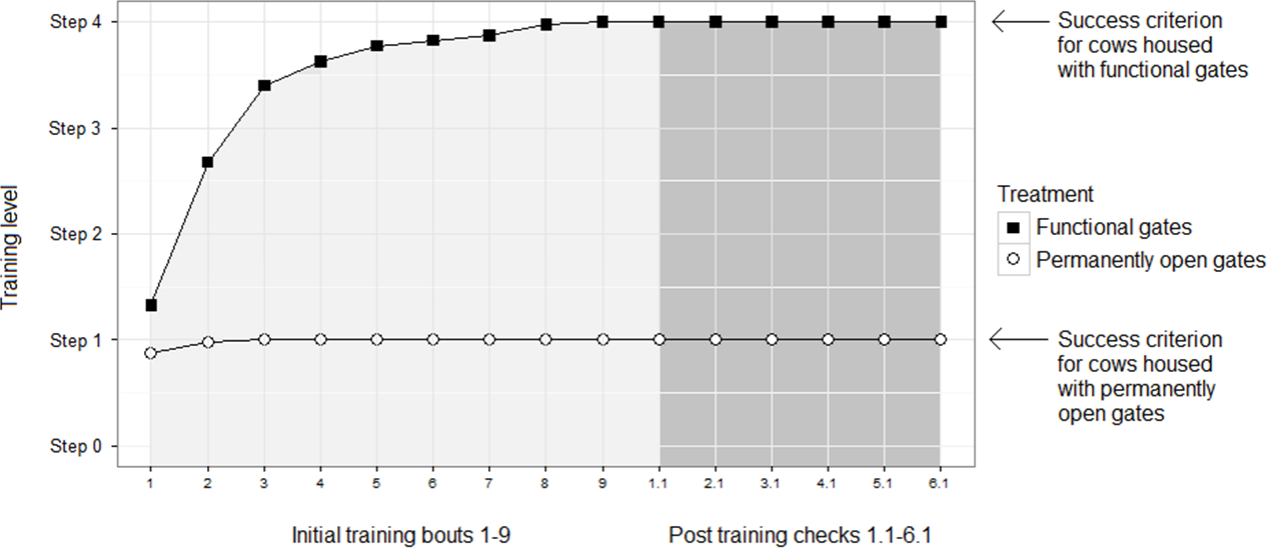
Graphs of mean training level per training bout for each treatment illustrating the progress during the initial training period (divided onto treatment: “Functional gates” and “permanently open gates”). Training level: Step 0 (not able to follow the trainer), step 1 (following the trainer in and out of individual calving pen = success criterion for “permanently open gates”) and step 4 (following the trainer in and out of individual calving pen while opening the gate without any help or encouragement = success criterion for “functional gates”). Training bouts 1–9 (2 bouts per day) leading to experimental start (shown in light grey) and subsequent weekly post training checks 1.1 to 1.6 (1 bout per day) during the experiment (shown in dark grey).

The training progress was supervised for all 78 cows to make sure all cows complied with the success criterion and to ensure that all were trained for approximately the same amount of time. Seventy-four cows reached the criterion after 4 successive days of training. However, four cows still showed strong fear reactions towards the trainer and the feed bucket upon their fourth training day (eighth training session). These were given two extra training days. Thus, median of training duration was 4 successive days (range 4 to 6 days) until compliance (Fig 4). One cow did not reach the success criterion (housed with functional gates) within these extra days, and was excluded from the experiment. The remaining three cows managed to reach the criterion and were included. Two cows calved during the training period (i.e. before complying with the criterion) and were excluded. In total, 75 cows; 41 housed with functional gates and 34 housed with permanently open gates, were included after the training sessions, and they all complied with the success criterion in the subsequent weekly post-training checks (Fig 4).

### Measures and analysis

#### Data editing and video recordings

For each cow the experimental period ended when she calved and each cow-calf pair was removed 5 to12 h after calving, in order to allow the cow time to nurse the calf and for the staff to have time to move them. This relatively rapid removal limited the period where potentially attractive alien calves were present [4]. Cows were excluded from the study if they had a stillborn calf (n = 2), gave birth to twins (n = 1), or had clinical milk fewer, mastitis or retained placenta (diagnosed by the herd veterinarian on the day of calving (n = 4)). Delivery of the calf was assisted if the calf was not born within 4 h after the appearance of the amniotic sac. Assisted calvings also resulted in the cow being excluded from the study (n = 2). Thus, 66 cows could be included in the analysis (35 housed with functional gates (from 7 groups having 5, 5, 5, 5, 4, 5, and 6 cows per group) and 31 housed with permanently open gates (from 6 groups having 4, 6, 5, 5, 6, and 5 cows per group); and 19 and 8 cows entering their second parity, 10 and 16 cows entering their third parity, and 6 and 7 cows entering fourth or later parities, respectively, for the two treatments). The behaviour of the cows was monitored by digital video cameras (model: TVCCD-624, Monacor, Bremen, Germany) mounted above the sections: Two cameras covered each end of each group area and one camera was mounted above each individual calving pen, leading to a total of 8 cameras per section. Recordings were made continuously throughout the whole experimental period, and an experienced technician monitored and stored all video material. The behaviour of each cow was observed continuously for 12 h prior to calving by an experienced observer after termination of the data collection. The same observer performed all video analyses.

#### Use of the individual calving pens and calving

Location of the cow and the occurrence of behavioural elements (Table 3) were monitored in order to keep track of the calving process and the use of the individual calving pens. The response variable was the location of the birth place (the placing of the cow when the hips of the calf were fully expelled from the birth canal), which also determined the end-point of the observation period. The location of the cow was determined as either within the group area or within an individual calving pen. If a cow was located between the two areas, the head of the cow determined the outcome. This definition was also used when determining where the cow experienced her first sequence of rhythmical abdominal contractions. The duration of the second stage of labour was defined as initiated at the first rhythmical abdominal contraction bout (Table 1) and continuing until the hips of the calf were fully expelled ([7,37]).

**Table 3:** Ethogram of continuously recorded behaviour during the 12-h period prior to calving.

*Placement of focal cow*	*Description*
In group pen	> 50% of the body placed in the group pen
In a single pen	> 50% of the body placed in a single pen
*Behaviour of focal cow*	*Description*
Displaced by another cow	The focal cow moves away, more than the length of a cow, after another cow has approached the focal cow rapidly, made a ‘head swing’ towards the focal cow or butted the focal cow’s head or body.
Displacing another cow	The focal cow causes another cow to move away with more than one cow length, by approaching the other cow rapidly, making a ´head swing´ towards the other cow or butting the other cow’s head or body.
In contact with the gate	The focal cow is in physical contact with the gate (head, neck and/or shoulder).
Locked gate mistrial	The focal cow places the neck over the gate and pushes the gate with no success of opening it. (Only entering an individual pen with a functional gate)
Missed entering/re-entering	The focal cow opens the gate fully or partially, enters the gateway, but then moves backwards again to the side from where she came, or the cow enters the gateway but then moves backwards to the side from where she came.
Start of rhythmical abdominal contractions	First time the abdominal muscles contracts and release repeatedly in a rhythmic motion, cow can be standing or lying[7].
*Other observations*	*Description*
Calf´s legs visible	Calf´s legs visible outside the vulva of the focal cow, legs may be covered by the amniotic sac[7].
Another cow is licking the calf legs	Another cow licks the legs of the calf before the calf is born.
*Behaviour terminating the observations*	*Description*
Calving	The hips of the calf are successfully expelled from the focal cow[7].

The event of calving determined the endpoint of the observation period and thus all cows were observed according to their placement and behaviour within the experimental section group 12 h prior to calving.

#### Social behaviour

Social behaviour includes displacements with physical contact and displacements without contact (described in Table 3). For each focal cow all displacements given and received were summarized over the 12 h video observation period. Furthermore, it was noted if an alien calf was present within the last 8 h before calving as this is the approximate period where cows have been reported to isolate [19,20]. Within each group, the order of calving was noted. For each calf born, a pregnant heifer (average age and body weight: 21.6 months and 575 ± 48 kg) was added to the group pen after the removal of the cow and her calf.

#### Gait scores and body weight

Cows were gait-scored when entering and exiting the experiment. The scoring was done by two experienced observers according to the 5-point scoring system developed by Thomsen et al. [38]. Two cows were scored as obviously lame (above score 3) when entering the experiment, but treated for laminitis (hoof washing followed by application of a bandage with salicylic acid treatment) upon entering, and scored 2 and 1, respectively, before calving. Median gait score was 1, when entering as well as exiting the experiment (range 1 to 4 at entry, and range 1 to 3 when exiting). Cows were weighted upon entering the experiment and when leaving the experiment 5 to12 hours after calving, by the use of the same automatic scale (Danvægt, Hinnerup, Denmark). The cows weighed on average (mean ± s.d) 687 ± 75 kg and 715 ± 61 kg when entering the experiment, and 674 ± 56 kg and 692 ± 65 kg when leaving the experiment, for cows housed with functional gates and for cows housed with permanently open gates, respectively. After calving, cows were machine milked once in the experimental pen before being moved from the barn. Rectal temperature was measured at this milking (mean ± s.d.: 38.7 ± 0.8°C).

### Maintenance of bedding and gates

Bedding quality of each individual calving pen and group area was evaluated daily at 08:30–10:00 am prior to removal of manure. The quality of the bedding was scored by the barn staff according to a 5-point scale [39]: (0) dry, no faeces or urea, (1) moist, some parts with faeces (n < 3), (2) slightly wet, both dark and light straw and more than 3 parts with faeces, (3) wet, mostly dark straw and faeces, (4) very wet, only dark straw and faeces and urea spread over the whole pen (median: 1, range: 0–4).

The force needed to open a functional gate from inside the individual calving pens and from the group area was measured by an electronic scale (model OCSF300, Scale House ®, 3711 NW 36th St., Miami, FL 33142) at the end of the experiment (5 measures per gate), showing that 12.5 ± 1.8 kg and 12.2 ± 3.6 kg force was needed, respectively.

### Statistical analysis

#### Behavioural variables

We calculated the social dominance as a so-called ‘rank ratio’ for each cow based on the displacements summarized over the 12 h before calving: the number of individuals being displaced by the focal cow, and the number of individuals displacing the focal cow; the higher the ratio the higher the rank [40]. Afterwards, the ratio was converted into an index (x 100) indicating if the cow was in the dominant or subordinate end of the scale from 1–100 within the group. Calving order was identified as a categorical variable with 6 levels. The presence of an alien calf (a calf born from another cow which was not yet removed before the next calving) during the last 8 hours before calving was categorized as one or zero (present or absent). Similarly, the location where the cow experienced her first sequence of rhythmical abdominal contractions was categorized as ‘Group area’ or ‘Individual Pen’. A preliminary analysis showed positive correlations (based on Mann-Whitney U test; [41]) between personality score (shy, bold) and dominance and thus only one of these two variables could be included in the model. Social dominance was chosen over personality assessment (‘shy’ or ‘bold’) as this measurement was based on observations of the cows close to time of calving.

Based on Shapiro-Wilk normality test and visual assessment of histograms, normality of the recorded variables could not be assumed. Hence, all variables were analysed using models and tests not assuming normality. Statistical analyses were performed using the R software, version 3.1.2 (R Core Team 2014, Vienna, Austria) and all p-values evaluated according to a significance level of 5% and 10% for tendencies.

#### Modelling

Due to the primary response variable having a binary outcome (i.e. calving site), data were analysed using logistic regression and package lme4 [42] in the R software. It was not possible to include season or other time-related measures in the analyses, as the section groups were temporally overlapping to ensure suitable inter-calving intervals within group. We did, however, include the placement of each section group within the barn as a random effect in a mixed effects model. Thus, the full model included the random effect of placement within barn (i.e. section number 1, 2 or 3 in Fig 1) as well as the fixed effects; dominance ratio within group at calving (mean ± s.d.: 64 ± 33), duration of second stage labour (mean ± s.d. (min): 108 ± 51), calving order within group (1, 2, 3, 4, 5 or 6), parity (second, third or older), presence of alien calf (0 or 1) and the location where the cow showed her first rhythmical abdominal contractions (individual pen, or group area). The full model did not show any effect of the random variable “section” (variance and s.d. = 0), which was subsequently excluded. A normal logistic regression model was then fitted to test effects of the explanatory variables. The normal logistic regression model was reduced using a backwards stepwise procedure with p < 0.1 as inclusion criteria. The final model included the fixed effects; rank ratio within group at calving, presence of alien calf and the location where the cow showed her first rhythmical abdominal contractions. The model fit was checked using the Hosmer Lemeshow Goodness of fit test [43]. Odds ratios and 95% confidence intervals were constructed from the final parameter estimates by back-transforming, applying the inverse logit function.

#### Calving location

In order to examine whether cows calved in random locations within each section, and thus were not affected by the location of previous calvings (as suggested by [31]), a 1-sample proportion test with continuity correction was used [44].

## Results

All cows displaced other cows but also received displacements from other cows, and thus the social dominance of each cow within each group could be determined through rank ratio calculation without any cows sharing the same level of dominance within a group. Bold cows (n = 34) had significantly higher rank ratios whereas shy cows (n = 32) had lower rank ratios (Mann Whitney U-test: (median; range (rank ratio index)) bold: 93; 6–100 vs. shy: 41; 0–100, W = 806, r = 0.8, p < 0.001).

### Factors affecting isolation seeking behaviour at calving

#### Effect of being housed with functional gates

Across the two treatments 34 cows calved in an individual calving pen and 32 in the group area. The odds of calving in an individual calving pen tended to increase when cows were housed in sections with permanently open gates compared to cows housed in sections with functional gates (Table 4).

**Table 4:** Summary of the output from the final model including 4 fixed effects.

Variable	Levels	No. of animals	*b*	S.E.(*b*)	OR	95% CI (OR)	*p*
Intercept			-10.25	3.75	-	-	
Treatment	Gate	35	0	-			0.068
	No gate	31	3.94	2.16	51.41	1.83; 72.97	
Rank ratio	Continuous	66	0.13	0.045	1.14	1.07; 1.30	0.0035
Presence of alien calf	No	39	0	-			0.069
	Yes	27	-4.67	2.58	0.46	0.011; 1.25	
1^st^ rhythmical abdominal contractions	Group area	32	0	-			0.036
	Individual calving pen	34	3.99	1.91	54.05	2.89; 162.82	

‘Treatment’ i.e. having a functional gate or a permanently open gate, ‘Rank’ i.e. the rank ratio index within each group at calving, ‘Presence of alien calf’ i.e. having an alien calf present within the last 8 h before calving or not and ‘where 1^st^ sequence of rhythmical abdominal contractions occurred’ i.e. in the group area or in an individual calving pen. Odds ratio for each variable with corresponding 95% confidence intervals and p-values are presented along with coefficients ‘*b*’ and stand errors ‘S.E.(*b)*’.

#### Effect of dominance

Rank ratio significantly affected choice of calving site; the higher the rank within a group at calving, the higher the odds of calving inside an individual calving pen. For every increase in rank order of 1 the odds of calving inside the individual pen increased by 1.14 (Table 4), and due to the assumption of proportional odds in the model this was the same for each level of rank e.g. rank 33 to 34 and rank 66 to 67.

#### Effect of location of first rhythmical abdominal contractions

Another factor, influencing the choice of calving site, was the location of the first sequence of rhythmical abdominal contractions. Fifty-one percent of the cows had the first sequence of rhythmical abdominal contractions while being in an individual calving pen and these cows had significantly higher odds of calving inside the individual calving pen than cows showing their first sequence of rhythmical abdominal contractions within the group area (Table 4). Additionally, 10 cows (4 housed with functional gates and 6 housed with permanently open gates) had their first rhythmical abdominal contraction in the group area and then moved to an individual calving pen during the second stage of labour, whereas 6 cows (3 cows housed with functional gates and 3 cows housed with permanently open gates) moved from an individual pen to the group area during the second stage of labour.

#### Effect of presence of an alien calf

An alien calf was present within the last 8 hours prior to another calving for 41% of the cows. For these cows the odds of calving in an individual calving pen decreased by 0.46 (Table 4).

Three variables had no effect in the full model and were thus stepwise removed from the model: First parity (p_parity2_ = 0.85 and p_parity3_ = 0.62), then duration of second stage labour (mean ± s.d.: 108.5 ± 51.4 min) (p = 0.65) and lastly calving order within group (1 to 6) (p_2_ = 0.89, p_3_ = 0.16, p_4_ = 0.49, p_5_ = 0.38 and p_6_ = 0.41). These had no effect on the odds of calving inside an individual calving pen (p > 0.1) and were thus not included in the final model.

### Calving location

The median dispersion between calvings within group was 6 (range: 0 to 20) days. For 8 out of 13 groups, one or two cows (n = 10) calved in close proximity to the location of the last calving (within one cow length from the site of last calving). When testing these groups separately in the 1-sample proportion test, however, no evidence for selection of calving site due to the site of a previous calving could be found, neither for groups with one cow calving close to another or groups having two cows calving close to a third cow.

## Discussion

In this study, we gave cows an opportunity to move to another pen to calve and examined whether isolation seeking behaviour was facilitated by this opportunity to isolate (seek seclusion as well as increase the distance to group members). Additionally, the study included social aspects of housing pre-parturient cows in groups and the effects of these on choice of calving site. We found that 50% of the cows moved away from the group and isolated in an individual calving pen regardless of the presence of a functional gate in the pen or not. Unexpectedly, cows housed with permanently open gates tended to be more likely to calve in the individual calving pens than cows with functional gates. Dominance increased the odds of calving in an individual calving pen whereas the presence of an alien calf, during the last 8 hours prior to calving, decreased the odds.

Contrary to the hypothesis, cows housed with permanently open gates tended to be more likely to calve in the individual calving pens compared to cows housed with functional gates. This implies that the cows experienced the gates as obstacles rather than an advantage for isolating before calving. As we have previously found that cows seeking maximum isolation (75% versus 50%) at calving had longer second stage labour [39], the fact that there was no effect of the duration of second stage labour on the choice of isolation in this study further supports that the gate may have been an obstacle for the cows. All cows received prior training and complied with a learning criterion for being able to use the gate before being included in the experiment, suggesting that the cows had learnt to use the gate. In none of the treatments did the choice deviate from random. In this study, the cows had to combine a learnt and rather conspicuous response (opening the gate and knowing that no one could follow) with the motivation to isolate. Research on the cognitive abilities of cattle is generally limited, but cows do possess the ability to perform rather complex instrumental conditioning tasks [45], have quite good spatial memory and are able to navigate quite complicated mazes which they memorize for up to 6 weeks [46]. Furthermore, all cows used the pens on their own several times before calving, and approx. 50% ended up calving in one of the pens. Therefore, there is no particular reason to argue that the cows did not learn and memorise the task of opening the gate. Memorising a conspicuous task compared to memorising e.g. a route to a preferred grass patch may however, be of less biological relevance (as argued for horses by [47]). Furthermore, being in labour and experiencing pain may have made it difficult for the cows to recall this knowledge. Not because the task of isolating was not biologically relevant, but because the task of opening a rather conspicuous gate was less biologically relevant in this situation. The gate may also have been perceived as too heavy to manoeuvre, or uncomfortable to pass, especially once the cow was in labour. Being in labour, the abdomen of a pregnant cow may become increasingly sore as contractions arise [1] and thus opening a gate which place pressure on the abdomen may be unpleasant. Furthermore, being in labour pain, cows may have tried to avoid potential conflicts with conspecifics and thus they may have favoured to be able to control their visual field and have the opportunity to withdraw from conspecifics. Therefore, entering a gated pen may have been too risky also because it required effort to leave. In terms of the evolutionary history of ungulates, the trade-off between isolation seeking and having less control in term of executing a flight response to avoid a predator also supports this. Lastly, the distance between the group area and the individual calving pen may have influenced whether cows used the individual pens for calving or not. In a semi-natural setting, Lidfors et al. [19] noted that cows walked a considerable distance away from the group and it is thus possible, that in the present study, the cows perceived the individual calving pens as situated too close (maximum 9 m away) to the rest of the group in order for the pens to be perceived as offering isolation.

Future studies on motivation-based calving facilities may examine whether gates that are less conspicuous and require less effort to open result in more cows calving in them. Potentially, a gate that closes behind the cow when entering the calving pen, without a need for pushing or manipulation may be more appropriate. Also combining a simpler and less heavy gate with a pen that offers a higher level of isolation could be one option to facilitate the use of individual calving pens. In addition, potential effects of distance between individual calving pens and the group-members should be examined. Lastly, more research on learning and memorising of more complex instrumental tasks in cattle is needed in order to understand the capacity of cattle to learn how to perform a task and recall it in the peri-parturient period, where other underlying motivations may be strong.

In the present study social order within the group influenced whether cows chose to calve in the individual calving pens (Table 4). When analysing the variable “rank ratio” an assumption of proportional odds (i.e. that the odds for each increase in rank by a factor 1 would be the same) had to be made, and thus this assumption should be kept in mind when the results are interpreted. Furthermore, when using the term “rank ratio” we had to assume that the social dominance in these groups was linear, which may or may not be the case. However, if groups of pre-calving cows are newly established, or dynamic, there is a risk of aggression due to the establishment of dominance relations [25], especially near defendable resources. Cows become maternally motivated due to hormonal change prior to calving (reviewed in [48]) and as calving approaches an individual calving pen may become a valued site. In the present study, dominant cows may have guarded the individual calving pens leaving them a higher chance of calving there. Also being dominant they were less likely to be displaced from the pens with permanently open gates. This suggestion may be supported by the finding that cows positioned inside an individual calving pen when the second stage of labour was initiated, had a significantly higher probability of calving in the pen. It is possible, that the choice of where to calve was already taken when the second stage of labour was initiated, and thus, if a cow had been guarding an individual calving pen, she was more likely to end up calving there. Moreover, cows that changed their position after initiation of the second stage of labour mainly changed from the group area to an individual calving pen (10 out of 16 cows). These cows were mainly dominant cows (8 out of 10) and all cows moving from an individual calving pen to the group area (n = 6) were subordinate (happened in sections of both experimental treatments, 3 from each), further implying that dominance continued to play a role, even during the second stage of labour. These findings have implications for the design of a motivation-based calving facility as in this case, the facility only succeeded in proving an isolation opportunity for the dominant cows even though there was always one individual calving pen per cow. More research is needed in order to outline how to account for dominance in a motivation-based calving pen design. Additionally, this highlights the role of the social hierarchy in a pre-calving group and points out that farmers may have to consider that some cows need more protection if they are to move away from the group at calving. Another, perhaps surprising result, in relation to this, is that social dominance (“rank ratio”) strongly correlated with the personality assessment made prior to the experiment and at least one week prior to calving. This correlation showed that bolder and more explorative cows were also the more dominant while shy cows were more subordinate. This information combined with the above results may be useful for farmers trying to identify cows needing assistance in order to enter individual calving pens and shy cows may even have to be moved manually. In this experiment most shy cows calved in the group area, and protection from competition for individual calving pens may be required. Personality assessment may be a practical tool to assess dominance and to identify cows that may have to be moved manually to an individual calving pen.

Another social aspect of pre-parturient groups, which farmers also need to be aware of, is the presence of alien calves in the group. In this study, the presence of an alien calf lowered the probability of the cows calving inside the individual calving pens, potentially because of an attracting effect of either the calf as such [4] or the amniotic fluids in the fur of the new-born [49]. Combining this knowledge with the described effects of dominance, these social aspects may make a motivation-based calving system difficult to apply unless subordinate cows are moved manually to an adjacent maternity pen.

The fact that not all cows calved in the calving pens does thus not imply a lack of learning or knowledge of this opportunity, but rather a combination of competition and motivational conflict. For a motivation-based calving facility to function, these issues have to be solved. As discussed above, improving or increasing the quality of the isolation opportunity is important especially when aiming to facilitate isolation seeking at the time where they are motivated to do so. Competition and distractions are problems that may not be easily solved, but if a cow moves to an isolated area before giving birth and if her calf is subsequently retained there, this would nonetheless decrease the level of competition and distractions in the environment of the cows. From this perspective, another possible approach may be to use the presumably attracting effect of birth fluids [31] or amniotic fluid at specific points inside the calving pens. As these substances have previously been shown to attract cows no earlier than 12 h prior to giving birth [49], this may be an option to attract only the cows that are close to calving and move them away from the herd before they (or their calves) become a distraction. Additionally, this attraction may function as a somewhat neutral stimuli for cows that are not yet close to calving.

## Conclusions

In this study we could not show that use of a functional gate combined with prior training facilitated isolation seeking, as measured by the use of individual calving pens at the time of calving. This may be due to the rather conspicuous instrumental task of opening the gate or because the cows were trying to avoid a potentially confined situation where executing a flight response may be difficult. Alternatively, the cows may not have been able to combine the learnt response with the motivation to isolate. Social factors had a strong influence on the odds of a cow calving inside an individual calving pen, with dominant cows having the highest odds of occupying an individual calving pen in the moment of calving and the presence of an alien calf reducing the likelihood of cows calving inside the individual calving pen. Therefore, social factors have implications for the functionality of a motivation-based system. More research is needed to fully outline the cognitive basis from which cows are able to learn and recall learnt responses and whether these responses are overruled by other motivations once calving is in progress.

## Supporting information

S1 Video Cow performing the last step in the training procedure for cows housed with functional gates. The video mimics the training situation as this particular cow is not an experimental cow. She was trained on randomly chosen pens and with the five other group members present. Prior to this take, the cow has been successful in all the previous training steps. At this stage the cow is trained to open the fully closed gate on her own and subsequently enter the pen to reach the reward from the trainer facing the cow from inside the pen/the group area (step 4 in the training procedure). The trainer ensured that no other cows followed the focal cow into the individual calving pen at any point.
(MP4)

S1 File Gate errors. (DOCX)

S2 File Data. The data sheet provided for this study includes all necessary information in order to recalculate the results of the study. The sheet has one horizontal line for each individual cow in the experiment and each cow has its own identification number (‘cow_number’). All subsequent columns has an explanation line on the top of the sheet and whenever data is not available for the particular cow this is marked with a ‘.’. Two variables are one/zero variables corresponding to 1 = “yes and 0 = “no”.
(XLSX)
