# Supplementary material for: The motivation-based calving facility: Social and cognitive factors influence isolation seeking behaviour of Holstein dairy cows at calving
Source: PLoS One. 2018 Jan 18;13(1):e0191128. doi: 10.1371/journal.pone.0191128 (PMC5773170; doi:10.1371/journal.pone.0191128)
Supplement: S1 File — (DOCX) [file pone.0191128.s002.docx]

For cows housed with functional gates, all technical errors related to the use of the gates were recorded (Table 3). Eight cows experienced a locked gate due to errors. The errors were either due to cows not fully entering the pen, and thus locking the gate without a cow inside (n=6), or due to erroneous locking of the gate (n=2). However, in all of these 8 cases, the cow re-entered another pen within 60 min after experiencing the error.
